# Innate Responses to Putative Ancestral Hosts: Is the Attraction of Western Flower Thrips to Pine Pollen a Result of Relict Olfactory Receptors?

**DOI:** 10.1007/s10886-014-0450-0

**Published:** 2014-05-31

**Authors:** Zayed S. Abdullah, Katherine J. Ficken, Bethany P. J. Greenfield, Tariq M. Butt

**Affiliations:** 1 Department of Biosciences, Swansea University, Singleton Park, Swansea, SA2 8PP UK; 2 Department of Geography, Swansea University, Singleton Park, Swansea, SA2 8PP UK

**Keywords:** *Frankliniella occidentalis*, Thrips, Pine pollen, Semiochemical, (S)-(-)-Verbenone, Electroantennogram, Insect pest

## Abstract

Pollinophagy is widely documented in the order Thysanoptera, with representative individuals from six of the nine divergent families known to feed on pollen. Various pollens of the genus *Pinus* increase the development time, fecundity, longevity, and settling preference of Western Flower Thrips (WFT), *Frankliniella occidentalis* (Pergande) (Thysanoptera: Thripidae). Certain species of flower thrips discriminate among pollen types, but no studies have elucidated the olfactory cues that play a role in their pollen preferences. In this study, the volatile organic compounds emitted by pollens of the genus *Pinus* were elucidated. Various chemicals from pollen headspace elicited electrophysiological responses from WFT antennae. The compound *(S)*-(-)-verbenone, identified in pollen headspace, attracted WFT in a 4-arm olfactometer. This compound has potential for use in integrated pest management programs against the pest. We present the hypothesis that this polyphagous insect may have retained ancestral ‘relict’ olfactory receptors through the course of evolution, to explain this attraction to pine pollen. This attraction has allowed the insect to find and exploit an unusual nutrient source that significantly increases its fitness. The study demonstrates how fossil record analysis and subsequent evolutionary knowledge can aid in explaining possibilities as to why some insects sense and respond to chemicals that would otherwise seem peculiar to their ecology, allowing insight into the evolutionary forces that may shape insect olfactory systems over time.

**Electronic supplementary material:**

The online version of this article (doi:10.1007/s10886-014-0450-0) contains supplementary material, which is available to authorized users.

## Introduction

Thrips (Thysanoptera) of two sub-orders, comprising six of the nine divergent families, supplement their diet with various pollens, a highly nutritious but non-essential food source (Heming 1993). Representative species from three families feed on pollen by using their forelegs to grab individual grains (Kirk 1984, 1985, 1987), a method of feeding that differs slightly from that used in feeding on plant tissue. This suggests that pollinophagy is either a primitive feature or that it has evolved independently in several families of Thysanoptera. There is fossil evidence of Mesozoic fossilized thrips carrying pollen of ancient gymnosperms (Penalver et al. 2012), consistent with pollinophagy being a primitive trait in the order.

Western Flower Thrips (WFT), *Frankliniella occidentalis* (Pergande) (Thysanoptera: Thripidae) is a major agricultural and horticulture pest worldwide (Kirk 2002; Kirk and Terry 2003). This insect causes damage and spoilage to a large number of economically important plant species through feeding, oviposition, and spread of several plant diseases, notably tospoviruses (Morse and Hoddle 2006). The cryptic nature and small size of this insect means that it can remain undetected through quarantine control measures. Its distribution throughout the world has been facilitated by the increase in international plant movement (Kiritani 2001). A major concern with this insect is the rapid development of insecticide resistance in populations (Bielza et al. 2007; Bielza 2008). This problem is expected to become more important because many pesticides have been withdrawn from use in the EU (Directive 2009/128/EC (2009)). This highlights the need to develop alternative control and monitoring methods.

Various pollens of the genus *Pinus* increase the development time, fecundity, longevity, and settling preference of WFT (Chitturi et al. 2006; Hulshof and Vanninen 2002). Separate studies suggest that WFT population dispersal may be positively correlated with pine pollen dispersal (Chitturi et al. 2006; Riley et al. 2007, 2011). Both pollen and the oily coating of pollen grains, known as pollenkitt, produce species-specific odors that can be distinguished by bees (Dobson 1987). Certain species of flower thrips have been shown to discriminate among host plant pollen (Kirk 1984, 1985), but no studies have elucidated the olfactory cues that play a role in this preference of thrips.

Our study is the first, to our knowledge, that has identified the volatile organic compounds (VOCs) emitted by pine pollen and tested what VOCs elicit both electrophysiological and behavioral responses in WFT. These chemicals could be incorporated into new integrated pest management strategies targeting WFT, as well as give us a better understanding of the olfactory capabilities of this pest.

## Materials and Methods

### Rearing

A colony of WFT, provided by Keele University, UK, was maintained on a bouquet of mixed cultivar chrysanthemum, *Dendranthema grandiflora*, potted in multi-purpose growing media (to allow for pupation of thrips), and kept on capillary matting inside ventilated Perspex cages (L 30 × W 30 × H 60 cm) at 25 ± 2 °C and L18: D6 photoperiod. The capillary matting was kept damp with distilled water. A fresh bouquet was placed in the rearing chamber every week, such that three bouquets were present in the rearing chamber at any one time. The oldest bouquet was removed upon addition of a new bouquet; this cycle provided optimal rearing conditions.

### Pollen

*Pinus elliotti* pollen was collected by members of the Department of Entomology, University of Georgia, near Athens, Georgia, USA. *Pinus sylvestris* and *Pinus massoniana* pollen was purchased from Amla Berry Ltd (New Delhi, India), and NatuHealth Ltd (Dundee, UK), respectively. All pollen samples were stored at −80 °C and used within one month of acquisition.

### Volatile Collections

Pollen volatiles were collected using static headspace Solid Phase Microextraction (SPME). Ten milligrams of each pine pollen sample were placed into specialized 300 μl glass inserts in 2 ml screw top vials (03-FISV, Chromacol Ltd, Herts, UK), capped with 9 mm silicone/polytetrafluoroethylene screw caps [9-SC(B)-ST1, Chromacol Ltd, Herts, UK]. Pollen volatiles were allowed to equilibrate in the headspace for 1 h at room temperature (ca. 24 °C) prior to collection. For collection of headspace volatiles, a 50/30 DVB/Carboxen^TM^/PDMS StableFlex^TM^ SPME fiber (Gray Fibre, Supelco) was inserted through the septum and exposed for 1 h. The fiber was desorbed in a gas chromatograph (GC) injection port within 5 min of retraction. Fibers were preconditioned as per the manufacturer’s guidelines, with a blank run always performed before adsorption so as to ensure the fibers were free of contamination. Three replicates were carried out in this fashion, using three batches of each of the three pollen samples. For standards, the same procedure was used depositing 1 μl of *n*-hexane containing 100 ng of test chemical into the same vials.

### Gas Chromatography/Mass Spectrometry

Gas chromatography/mass spectrometry (GC/MS) analysis was carried out on an HP6890 gas chromatograph coupled to a 5975 inert Mass Selective Detector (Agilent Technologies) operated in electron impact ionization (EI) mode (at 70 eV). SPME fibers were inserted into the GC split/splitless injection port (at 230 °C), fitted with a Merlin Microseal (Thames-Resteck, High-Wycombe, UK), and operated in splitless mode. A 2 min desorption time was allowed. The GC was fitted with an HP-5MS (J and W Scientific) fused silica capillary column (30 m × 0.25 mm × 0.25 mm film thickness). The oven temperature was held at 40 °C for 2 min and then increased by 10 °C.min^−1^ to 250 °C. Helium was used as carrier gas. Tentative identifications were made by comparing spectra with those of authentic samples in two separate mass spectral databases: NIST, 2011 version and Wiley 9th edition. Authentic standards, if available, were used to confirm our tentative identifications (although we did not identify specific enantiomers). Blanks were run of empty vials to eliminate background peaks. In order to determine which chemicals the thrips had been exposed to in the rearing chamber, volatile collections of the headspace of fresh chrysanthemum bouquets also were analyzed.

### Chemicals

The purities and sources of chemicals used in this study are listed in Table 1.

**Table 1:** Sources and purities of authentic chemical standards

Chemical	Source	Purity
(-)-borneol	Sigma Aldrich	97 %
(-)-bornyl acetate	Sigma Aldrich	95 %
(-)-caryophyllene oxide	Sigma Aldrich	≥99 %
(-)-*trans*-caryophyllene	Sigma Aldrich	≥98.5 %
(+)-longifolene	MP Biomedicals	Analytical Standard
*(1S)*-(-)-β-pinene	Sigma Aldrich	99 %
*(S)*-(-)-camphor	Fluka	≥99 %
*(S)*-(-)-verbenone	Sigma Aldrich	≥93 %
*(Z)*-pinocarveol	Sigma Aldrich	≥96 %
*(1R)*-α-pinene	Sigma Aldrich	98 %
*(1R)*-(-)-myrtenal	Sigma Aldrich	98 %
acetic acid	Fluka	≥99.5 %
ethyl acetate	Fisher Scientific	HPLC Grade
γ-butyrolactone	Sigma Aldrich	≥99 %
humulene (α-caryophyllene)	Sigma Aldrich	≥96 %
isoamyl alcohol (3-methyl-1-Butanol)	Sigma Aldrich	≥98 %
(-)-terpinen-4-ol	Sigma Aldrich	≥95 %
α-terpineol	Sigma Aldrich	≥96 %
*(Z)*-3-hexen-1-ol	Sigma Aldrich	97 %

### Electrophysiological Recordings of Adult Female WFT Antennae

Electroantennograms (EAGs) of the antennae of WFT to pollen VOCs were carried out as follows. The anterior portion of an adult female WFT was excised at the groove at which the mesonotum connects to the pronotum, and both antennae excised at the groove between antennal segments 7–8 (most distal to the head) to allow for better contact with the electrodes. The preparation was mounted by placing the most proximal end into the reference electrode and both antennae into the recording electrode, with the aid of micromanipulators. The glass electrodes were filled with glucose-free Ringer’s solution (Maddrell 1969). Authentic standards (in 10 μl of hexane) were applied to strips of filter paper at a dose of 1 mg. A filter paper was placed in a Pasteur pipet and allowed to sit for at least a minute to allow the solvent to evaporate. The pipet was then attached to a 5 ml syringe *via* rubber tubing. The syringe plunger was depressed (over less than 2 sec), expelling the volatile contents of the pipet into a purified airstream at a flow of 1 l.min^−1^ through a glass tube (i.d. 120 mm) over the preparation. Separate syringes were used for each chemical. This method gave consistent EAG peaks using a standard of *(Z)*-3-hexen-1-ol (see Fig. S1a in the electronic supplementary material). The EAG equipment consisted of a 10× gain universal probe (Syntech, Netherlands) and an IDAC 2 Signal Acquisition Processor (Syntech). Data were analyzed with EAGPro Version 2 software, (Syntech). *(Z)*-3-Hexen-1-ol standard was tested at the start of each replicate, and these responses were used to normalize all test recordings to the largest response recorded in an experiment. Responses to a hexane control were measured at the start and end of each preparation; these were normalized and averaged. Nine recordings were made to each test compound using a minimum of three insects. Data were square root transformed for homogeneity of variance, and analyzed using a general linear model. The model incorporated fixed effects of treatment, with insect specimen used for each recording included as a random factor. Means were compared using a Dunnet’s *post-hoc* test (comparing treatments to the hexane control) with SPSS software (IBM Corporation, USA).

### Bioassays of Adult Female WFT to Pine Pollen, Chrysanthemum Bud and Chemicals

A Perspex four-arm olfactometer (Pettersson 1970), with an arena diameter (arm to opposite arm) of 120 mm was used to determine behavioral responses of adult female WFT to whole pollen samples and to chemicals identified in pollen headspace. Samples were placed in 14 mm i.d. glass arms with an i.d. of 3 mm at the connecting point. Prior to any experiment, all Perspex components of the olfactometer were washed with Teepol detergent (SupplyTrade Ltd, Kent, UK), rinsed with 75 % ethanol, followed by distilled water, and then left to dry overnight. All glassware was washed with Teepol detergent, rinsed with acetone, followed by water and heated overnight at 400 °C. The olfactometer was placed in a cardboard box (20 cm high) lined with thick black plastic sheeting to exclude visual cues. The light intensity was measured at 400 lux at the top of the box using a digital light meter (Model: LX1010B, Sinometer Instruments, China). The UV intensity was measured at 0.2 μW/cm^2^ using a UVA meter (Tecpel 830, Tecpel Co. Ltd, Taiwan). Three of the olfactometer arms were used for controls and the fourth for a treatment. The arms contained glass wool in their tips to prevent insects escaping, as well as to block any external visual cues. A filter paper disc (Whatman™ 1 Qualitative), for traction, lined the base of the olfactometer, as well as providing good contrast by which to observe the insect. Both pollen and standard chemicals were bioassayed. When testing pollen, 10 mg were placed on a 1 cm^2^ filter paper piece in the treatment arm, and blank filter paper pieces were placed in each control arm. For chemical standards, 10 μg of chemical (in 10 μl of hexane) were applied to a 1 cm^2^ filter paper piece; controls consisted of 10 μl of hexane applied to filter paper pieces. A positive control consisted of a chrysanthemum bud in the treatment arm and blanks in the control arms. The bioassay was started 5 min. after introduction of the samples into the olfactometer, to allow for a steady release of VOCs.

Adult female WFT were conditioned in a Petri dish containing a small piece of wet filter paper for at least 4 h prior to the start of an experiment. An individual thrips was placed in the top central hole of the olfactometer using a dampened fine tip paintbrush; a piece of glass tube was fitted into this hole and sealed with a piece of Blu-Tack (Bostik, Paris, France). The glass tube was connected to a rotameter (Supelco, UK) and air drawn through the central hole at a rate of 400 ml.min^−1^ using a 12 V rotary vane pump (Gardner Denver Ltd, Hampshire, UK). The olfactometer was divided into four equal sections. A thrips was given 1 min to acclimate to the olfactometer, before the total time it spent in the treated section was recorded over 16 min. During the experiment, the olfactometer was rotated every 4 min. Twelve replicates were carried out per treatment. Time spent in the treated section was analyzed using SPSS (IBM corporation, USA). Time spent in the treated region was converted to a percentage (of the 16 min.), logit transformed and compared to a test mean of −1.099 (logit transformation of 25 %) using a one sample *t*-test; 25 % being the time an insect would be expected to spend in a region randomly. A significantly higher amount of time in the treated region was interpreted as an insect showing attraction to a test odor, while a statistically lower amount of time in the treated region was interpreted as an insect showing avoidance.

## Results

### Volatile Compounds in Headspace of Pine Pollen

GC/MS analysis of the headspace of pollen from the three *Pinus* species (Tables S2a-c in the electronic supplementary material) showed that all three released α-pinene, β-pinene, borneol, β-caryophyllene, bornyl acetate, and verbenone. Terpinen-4-ol and α-terpineol were found in the headspace of *P. massoniana* and *P. elliotti*, while caryophyllene oxide, longifolene, and acetic acid were found in the headspace of both *P. sylvestris* and *P. massoniana*. Ethyl acetate, isoamyl alcohol, camphor, and α-humulene were found only in the headspace of *P. elliotti*, while pinocarveol, myrtenal, and γ-butyrolactone were detected only in the headspace of *P. massoniana* pollen. β-Pinene, camphor, borneol, bornyl acetate, β-caryophyllene, and caryophyllene oxide were detected in the headspace of both pine pollen and fresh chrysanthemum bouquets (Fig. S2a-d shows total ion chromatograms and overlays).

### Electrophysiological Responses of WFT to Volatile Compounds from Pine Pollen

**Fig. 1:**
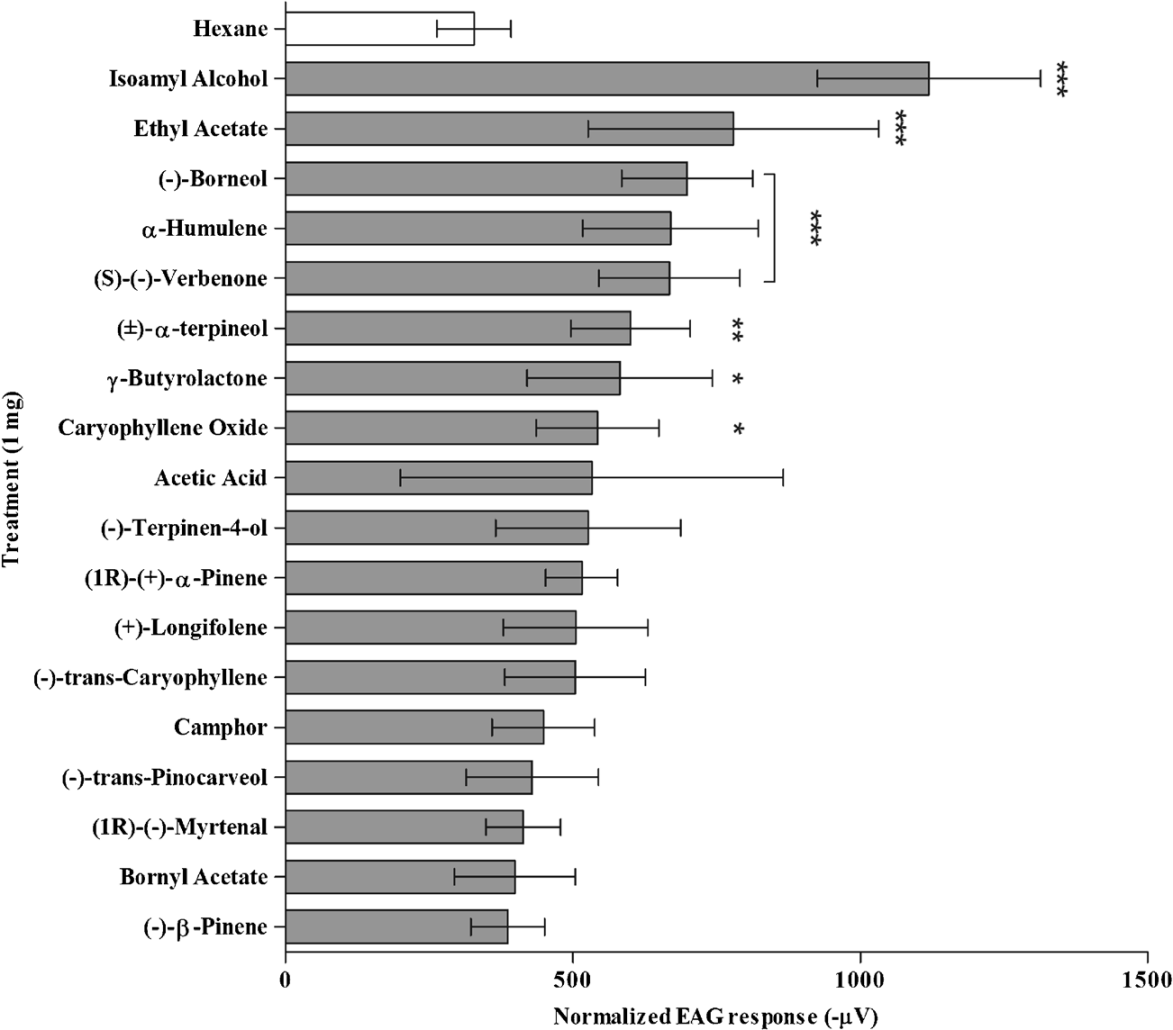
Mean electroantennogram (EAG) responses (normalized to *(Z)*-3-hexen-1-ol) of adult female western flower thrips to compounds identified from three pine pollen samples (*N* = 9) (data presented as mean ± 95 % C.I., ***-*P* < 0.001, **- *P* < 0.01, * - *P* < 0.05, compared to *n*-hexane control; general linear model, Dunnet’s *post-hoc* test)

Significant (different from the *n*-hexane control) EAG responses of WFT (Fig. 1) were recorded to isoamyl alcohol, ethyl acetate, (-)-borneol, α-humulene, and *(S)*-(-)-verbenone (all *P* < 0.001), (±)-α-terpineol (*P* < 0.01), and γ-butyrolactone and caryophyllene oxide (both *P* < 0.05). All other chemicals tested showed EAG responses that were not different to that from the *n*-hexane control. For representative EAG recordings of thrips to chemicals detected in pine pollen, see Figure S1b-t.

### Behavioral Responses of WFT to Pine Pollen and Individual Compounds

**Fig. 2:**
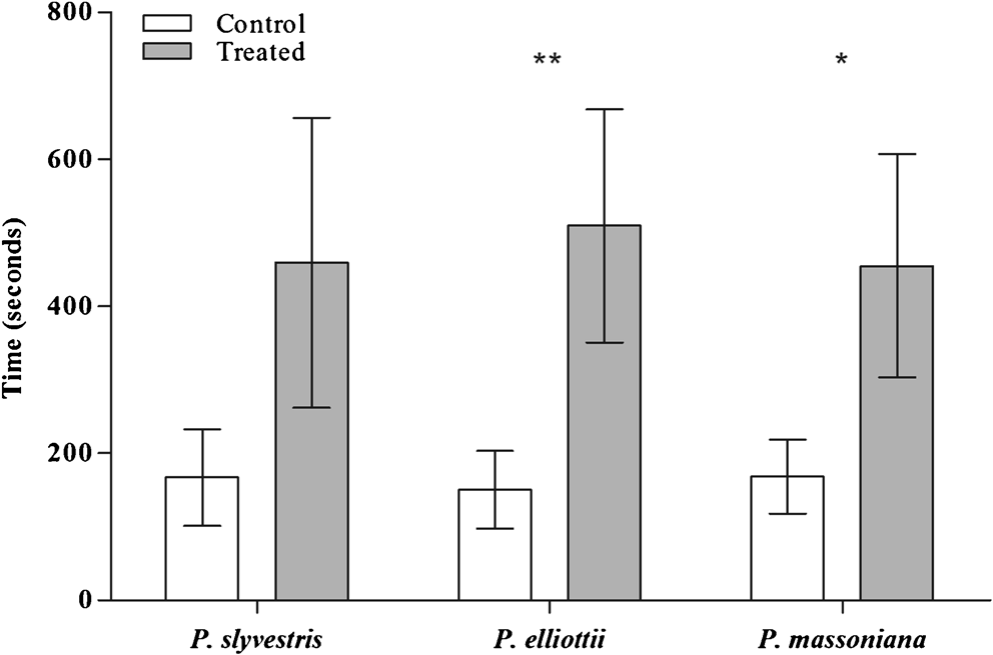
Mean responses of adult female western flower thrips to pollen odors from different *Pinus* species in a four-arm olfactometer. Time spent in the treated section was converted to a percentage of overall time, logit transformed, and compared to a test mean of -1.099 (logit transformation of 25 %) by a one-sample *t*-test. Data presented as ±95 % C.I., **- *P* < 0.01, *- *P* < 0.05), *N* = 12

**Fig. 3:**
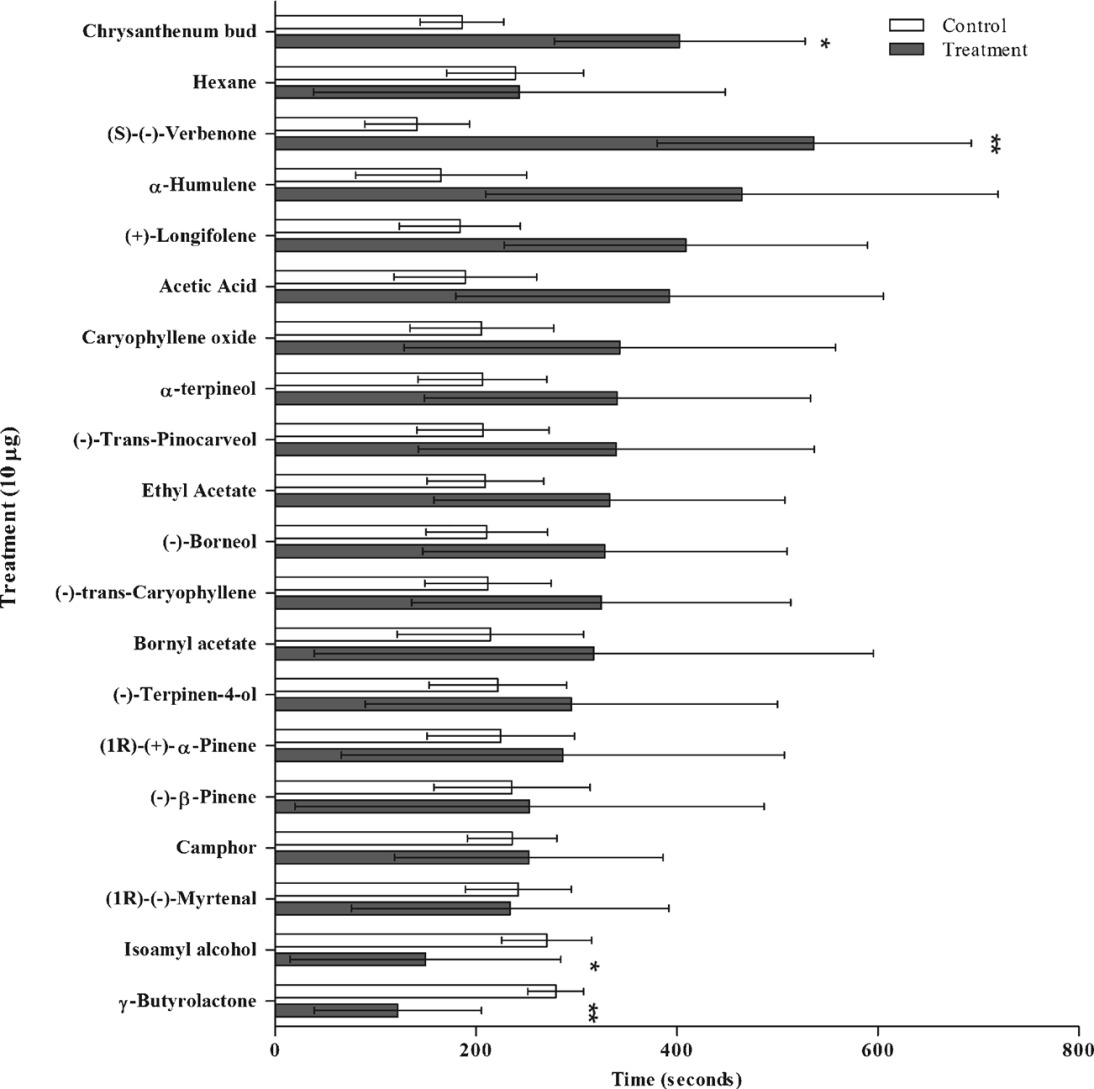
Mean responses of adult female western flower thrips to different odors in a four-arm olfactometer. Time spent in the treated section was converted to a percentage of overall time, logit transformed, and compared to a test mean of -1.099 (logit transformation of 25 %) by one-sample *t*-test. Data presented as ±95 % C.I., **- *P* < 0.01, *- *P* < 0.05, *N* = 12

WFT showed attraction to odors of *P. massoniana* (*P* = <0.05) and *P. elliotti* pollen (*P* < 0.01), but not to *P. sylvestris* pollen (Fig. 2). Of the individual chemicals, WFT showed attraction only to *(S)*-(-)-verbenone (*P* < 0.01). WFT exhibited avoidance to isoamyl alcohol (*P* < 0.05) and γ-butyrolactone (*P* < 0.01). WFT showed attraction to odors of a chrysanthemum bud (positive control), but not to the blank *n*-hexane (Fig. 3).

## Discussion

Pollen of *P. elliotti, P sylvestris,* and *P. massoniana* had similar VOC headspace profiles, consisting mostly of terpenoids, with few compounds unique to any one species. That that pines diverged from the closely related sister genus *Picea* some 87–193 million years ago (Grotkopp et al*.* 2004), and yet the three pine pollen samples had similar VOC profiles, suggests a relatively stable rate of odor evolution within the genus *Pinus*, at least with regards to the synthesis of pollen terpenoids. Compounds unique to specific pine species may be a result of environmental stress factors (*e.g.*, humidity, temperature, light, and nitrogen availability), which are known to evoke variability in volatile emission profiles among plants of the same species (Gouinguené and Turlings 2002). Pollen volatiles are chemically diverse compounds that may play multiple roles, and their production and emission may have evolved under conflicting selective pressures that protect the male gametophyte but also encourage dispersal by insects (Dobson and Bergstrom 2000). Several terpenoids (*e.g.*, caryophyllene, longifolene) detected in the headspace of pine pollen are known to have antibiotic properties and may protect against pathogens (De Moraes et al. 2001; Friedman et al. 2002; Hammer et al. 2003).

WFT are attracted to crude pine pollen (Chitturi et al*.* 2006), and certain flower thrips can discriminate among pollen of different plant species (Kirk 1984, 1985). Our study shows, for the first time, that specific olfactory cues mediate the attraction of WFT to pine pollen. Of the identified compounds in pine pollen, WFT showed electrophysiological responses and attraction to *(S)*-(-)-verbenone, suggesting that it is a major component responsible for attraction and perhaps preference (over other pollens) to pine pollen. This compound was released by pollen of all three *Pinus* species. *(S)*-(-)-Verbenone has not been widely reported as a semiochemical. It is known to repel certain coleopterans and attract others in coniferous forests (Paine and Hanlon 1991; Payne and Billings 1989; Progar 2005; Schlyter et al. 1989; Shea et al. 1992; Shore et al. 1992; Tilden and Bedard 1988). Headspace analysis of the chrysanthemums on which the insects were reared showed no trace of *(S)*-(-)-verbenone; hence, the response of WFT to this compound is likely innate.

Such an innate response to *(S)*-(-)-verbenone may have evolved in the last 40–50 million, a period during which most of the taxa that comprise the recent fauna of the order Thysanoptera evolved, presumably coinciding with the radiation of angiosperms (Grimaldi et al. 2004). Alternatively, such genetic refinement and development of a chemoreceptor may have evolved during the pre-angiosperm Mesozoic period, when gymnosperm-insect co-evolution was at its peak (Labandeira 2006), and has been retained as what could be termed a ‘relict’ olfactory receptor: a receptor that carries out secondary functions in extant species that were once involved with primary functions in their ancestors. We speculate that gymnosperm plants may have then been primary hosts to the ancestors of WFT. Clearly, individuals that are able to locate the most nutritious food sources, whether pollen or plant parts, would have a competitive advantage and concomitantly associated alleles would be conserved over evolution. Evidence to support such an idea could be provided by single-sensillum studies on the specificity of specific olfactory receptor neurons (ORNs) to *(S)*-(-)-verbenone and other structurally related compounds. In addition, studies on the presence of *(S)*-(-)-verbenone-specific ORNs in species across many families of Thysanoptera may confirm whether a ‘relict’ olfactory receptor hypothesis is valid or not, as convergent evolution in many distinct species in a relatively short period of evolutionary time would be unlikely.

While most compounds identified in the headspace of pine pollen either elicited attraction [*(S)*-(-)-verbenone] or neutral responses from WFT, isoamyl alcohol and γ-butyrolactone appeared to repel WFT. These compounds are produced during anaerobic respiration by microorganisms and can often be detected during fermentation (Pietruszka et al. 2010; Vose et al. 2001). Thus, these compounds may be produced by microbes, rather than by the pollen. We speculate that WFT may utilize these compounds to determine the quality of nutrient sources; *i.e.*, by avoiding food sources that are highly contaminated with microbes. The fact that WFT were attracted to pine pollen, even though these compounds were present, suggests that the ratio of attractants to repellents favored attraction. This is not unusual; many plants produce both attractant and repellent compounds, and it is the quantity and ratio or release rates that ultimately influence behavioral responses (Bruce et al*.* 2005). Alternatively, we may have tested these compounds at release rates much higher than that released naturally by pine pollen.

To summarize, we have shown that polyphagous WFT can detect and respond to volatiles from non-host species, which they may utilize as a supplementary nutrient source. Our research has identified compound(s) that could be used for monitoring or control of this important pest, and has demonstrated the value of researching non-primary hosts for novel semiochemicals.

## Electronic supplementary material

Below is the link to the electronic supplementary material.

ESM 1 (DOCX 4127 kb)

ESM 2 (DOCX 254 kb)
