# Supplementary material for: Innate Responses to Putative Ancestral Hosts: Is the Attraction of Western Flower Thrips to Pine Pollen a Result of Relict Olfactory Receptors?
Source: J Chem Ecol. 2014 May 31;40(6):534–40. doi: 10.1007/s10886-014-0450-0 (PMC4090808; doi:10.1007/s10886-014-0450-0)
Supplement: Supplementary file 1 — (DOCX 4127 kb) [file 10886_2014_450_MOESM1_ESM.docx]

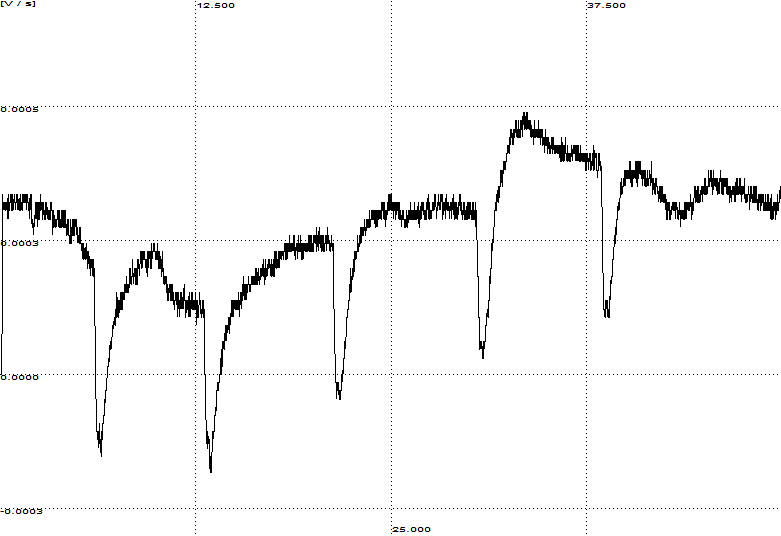


10µl of 1mg (Z)-3-Hexen-1-ol

10µl of 1mg (Z)-3-Hexen-1-ol

10µl of 1mg (Z)-3-Hexen-1-ol

10µl of 1mg (Z)-3-Hexen-1-ol

10µl of 1mg (Z)-3-Hexen-1-ol

**Figures S1b-t. Representative Electroantennogram responses for Standard (Green Line) and pine pollen volatile (Red Line)**

**b. (1R)-α-Pinene**

**c. (1S)-(-)-β-Pinene**

**d. (1R)-(-)-Myrtenal**

**e. (-)-Borneol**

**f. (-)-Terpinen-4-ol**

**g. (-)-Trans-Caryophyllene**

**h. (Z)-Pinocarveol**

**i. α-Terpineol**

**j. (+)-Longifolene**

**k. (S)-(-)-Verbenone**

**l. Acetic Acid**

**m. Humulene (α-caryophyllene)**

**n. (-)-Bornyl Acetate**

**o. (S)-(-)-Camphor**

**p. (-)-Caryophyllene oxide**

**q. Ethyl acetate**

**r. γ-butyralactone**

**s. Isoamyl alcohol**

**t. Hexane**
