# Supplementary material for: Innate Responses to Putative Ancestral Hosts: Is the Attraction of Western Flower Thrips to Pine Pollen a Result of Relict Olfactory Receptors?
Source: J Chem Ecol. 2014 May 31;40(6):534–40. doi: 10.1007/s10886-014-0450-0 (PMC4090808; doi:10.1007/s10886-014-0450-0)
Supplement: Supplementary file 2 — (DOCX 254 kb) [file 10886_2014_450_MOESM2_ESM.docx]

**Electronic Supplementary Material 2**

**Compounds detected in pine pollen headspace**

| Compound Number | Compound Name | Estimated amount in 10 mg Pollen Headspace (ng) | | | Ratio to the least detected compound |
| --- | --- | --- | --- | --- | --- |
| 1 | Ethyl acetate | | 1643 ± 503 | 54766 | |
| 2 | Isoamyl alcohol | | 211 ± 87.7 | 7033 | |
| 3 | α-Pinene | | 71.3 ± 27.5 | 2377 | |
| 4 | β-Pinene | | 37.1 ± 15.2 | 1237 | |
| 5 | Sabinene* | | - | - | |
| 6 | Camphor | | - 1. 0.859 |  | |
| 7 | (-)-Borneol | | 0.50 ± 0.066 | 16.6 | |
| 8 | Isocamphopinone* | | - | - | |
| 9 | Terpinen-4-ol | | 0.07 ± 0.017 | 2.33 | |
| 10 | α-Terpineol | | 0.03 ± 0.012 | 1 | |
| 11 | 3,6,6-TrimethylNorpinan-2-one* | | - | - | |
| 12 | Verbenone | | 1.3 ± 0.369 | 43.3 | |
| 13 | (-)-Bornyl Acetate | | 0.26 ± 0.091 | 8.67 | |
| 14 | β-Bourbonene* | | - | - | |
| 15 | β-Caryophyllene | | 1.39 ± 0.28 | 46.3 | |
| 16 | Humulene (α-Caryophyllene) | | 0.26 ± 0.045 | 8.67 | |
| 17 | γ-Muurolene* | | - | - | |
| 18 | α-Muurolene* | | - | - | |
| 19 | γ-Muurolene* | | - | - | |
| 20 | δ-Cadinene* | | - | - | |

**Table S2a** Compounds and estimated amounts (ng) identified in *Pinus* *elliotti* pollen headspace. Amounts were estimated using solid phase microextraction. * indicates that identification was not confirmed using an authentic standard. Highlighted compounds were also detected in the headspace of the rearing host plant *Chrysanthemum grandiflorum*.

| Compound Number | Compound Name | Estimated amount in 10 mg Pollen Headspace (ng) | Ratio to the least detected compound |
| --- | --- | --- | --- |
| 1 | Acetic Acid | 6915 ± 1841 | 691500 |
| 2 | γ-Butyrolactone | 319 ± 32.1 | 31900 |
| 3 | α-Pinene | 61 ± 5.78 | 6100 |
| 4 | Verbenene* | - | - |
| 5 | β-Pinene | 31.7 ± 2.91 | 3170 |
| 6 | β-Phellandrene* | - | - |
| 7 | Pinocarveol | 0.41 ± 0.029 | 41 |
| 8 | Unidentifiable* | - | - |
| 9 | Isocamphopinone* | - | - |
| 10 | Pinocarvone* | - | - |
| 11 | Borneol | 0.33 ± 0.024 | 33 |
| 12 | Isocamphopinone* | - | - |
| 13 | Terpinen-4-ol | 0.03 ± 0.006 | 3 |
| 14 | α-Terpineol | 0.01 ± 0.001 | 1 |
| 15 | Myrtenal | 0.37 ± 0.107 | 37 |
| 16 | Verbenone | 1.01 ± 0.025 | 101 |
| 17 | Bornyl acetate | 1.12 ± 0.03 | 112 |
| 18 | Longifolene | 0.21 ± 0.016 | 21 |
| 19 | β-Caryophyllene | 0.27 ± 0.027 | 27 |
| 20 | (-)-Caryophyllene oxide | 0.10 ± 0.005 | 10 |

**TableS2b** Compounds and amounts (ng) identified in *Pinus* *massoniana* pollen headspace. * indicates that identification was not confirmed with an authentic standard. Highlighted compounds were also detected in the headspace of the rearing host plant C*hrysanthemum grandiflorum*.

| Compound Number | Compound Name | Estimated amount in 10mg Pollen Headspace (ng) | Ratio to the least detected compound |
| --- | --- | --- | --- |
| 1 | Acetic Acid | 273.661 ± 122.577 | 3854.38 |
| 2 | α-Pinene | 0.416 ± 0.048 | 5.86 |
| 3 | β-Pinene | 0.213 ± 0.025 | 3 |
| 4 | Pinocarvone* | - | - |
| 5 | Borenol | 0.212 ± 0.062 | 2.99 |
| 6 | Verbenone | 1.183 ± 0.248 | 16.66 |
| 7 | Bornyl Acetate | 0.418 ± 0.077 | 5.89 |
| 8 | Longifolene | 0.109 ± 0.018 | 1.54 |
| 9 | Caryophyllene | 0.071 ± 0.019 | 1 |
| 10 | (-)-Caryophyllene Oxide | 0.114 ± 0.017 | 1.6 |

**Table S2c** Compounds and estimated amounts (ng) identified in *Pinus* *sylvestris* pollen headspace. * indicates that identification was not confirmed with an authentic standard as the chemical could not be sourced. Highlighted compounds were also detected in the headspace of the rearing host plant *Chrysanthemum grandiflorum*.

**Total Ion Chromatograms**

Figure S2a- Total ion chromatogram of *Pinus elliottii* headspace and blank septum vial.

Figure S2b- Total ion chromatogram of *Pinus sylvestris* headspace and blank septum vial.

Figure S2c- Total ion chromatogram of *Pinus massoniana* headspace and blank septum vial.

Figure S2d- Chromatogram overlay of *Chrysanthemum grandiflorum* headspace TIC, blank desiccant jar TIC and pine pollen TIC with (S)-(-)-verbenone peak indicated.
